# A Comprehensive Review of Bradykinin-Induced Angioedema Versus Histamine-Induced Angioedema in the Emergency Department

**DOI:** 10.7759/cureus.32075

**Published:** 2022-11-30

**Authors:** Maleesha Jayasinghe, Dilushini Caldera, Omesh Prathiraja, Rahul Jena, James Anwar Coffie-Pierre, James Agyei, Minollie Suzanne Silva, Abdul Mueez Alam Kayani, Ozair S Siddiqui

**Affiliations:** 1 Medicine, Nanjing Medical University, Nanjing, CHN; 2 Internal Medicine, Nanjing Medical University, Nanjing, CHN; 3 Medicine and Surgery, Nanjing Medical University, Nanjing, CHN; 4 Neurology/Internal Medicine, Bharati Vidyapeeth Medical College/Bharati Hospital, Pune, IND; 5 Orthopedics, Komfo Anokye Teaching Hospital, Kumasi, GHA; 6 Medicine and Surgery, Allama Iqbal Medical College, Lahore, PAK; 7 Medicine, Gujarat Medical Education and Research Society (GMERS) Medical College and Hospital, Patan, IND

**Keywords:** bradykinin-mediated angioedema, ace inhibitors, emergency, emergency department, bradykinin, bradykinin-induced angioedema, histamine, histamine-induced angioedema, angioedema

## Abstract

Angioedema (AE) is a condition that is frequently encountered in the emergency department (ED). It is a rare condition with localized, asymmetrical swelling of the skin and/or mucosa that is frequently nonpruritic and primarily affects locations with loose connective tissue. Physicians must have a thorough understanding of this condition since it can cause fatal airway compromise, which might be the presenting symptom. Histamine-mediated AE is the most common type of AE seen in EDs. However, ED physicians must be on the lookout for the less common bradykinin-mediated types of AE as these do not respond to the same therapy as histamine-mediated AE. Hospitals may lack specialized drugs or protocols, and many ED staff may be unable to identify or treat bradykinin-mediated AE. It is crucial to understand the pathophysiology of the various kinds of AE in order to optimize treatment. The goal of this review paper is to provide an overview of the pathophysiology, clinical manifestations, and treatment options for bradykinin and histamine-induced AE in the ED.

## Introduction and background

Angioedema (AE) is a sudden, non-pitting, transitory swelling of the skin, mucous membranes, or both, including the upper respiratory and gastrointestinal tracts, which generally lasts between several hours and three days [1]. AE tends to be asymmetrical and can occur anywhere on the body. However, the sites of predilection include the face, lips, mouth, throat, larynx, extremities, genital area, and gastrointestinal tract [2,3]. Lip and eye (periorbital) swelling are the most common. The upper airway swelling, primarily laryngeal edema, can be potentially life-threatening, but extreme pharyngeal and tongue swelling can also be disastrous [1]. After asthma, AE is the most prevalent cause of hospitalization among all allergic disorders [4]. Different types of AE are hard to distinguish in the emergency department (ED) setting. As no validated tests are available to distinguish between bradykinin-induced AE quickly, differences in the typical clinical presentation can help guide a diagnosis and be life-saving as these types of AE respond to specific treatments [4-7].

A detailed clinical history and physical examination can determine different subtypes of AE. However, in approximately one-third of hospitalized patients with AE, no cause can be determined; these individuals are classified as having idiopathic AE [4,6]. The most commonly encountered AE in daily practice is histaminergic AE [8]. Histamine-induced AE may present with urticaria and other manifestations of anaphylaxis, such as bronchospasm, wheezing, and hypotension. Urticaria affects the skin's surface layers, and fluid extravasation into the interstitial spaces causes swelling in the dermis and subcutaneous tissues. Generally, the onset is rapid, and the attack duration is brief [4]. Urticaria usually rules out bradykinin-mediated hereditary, acquired, and drug-induced AE (DAE) diagnoses [4]. On the other hand, bradykinin-induced AE is much less commonly encountered and typically presents with a slower onset, longer duration, and involves abdominal symptoms [5].

When diagnosing AE, differentiating bradykinin-mediated AE from histamine-mediated AE remains a recurring difficulty for physicians. Despite being less common than histamine-mediated AE, bradykinin-mediated AE is responsible for the bulk of the significant morbidity and mortality associated with AE. Therefore, appropriately diagnosing bradykinin-mediated AE when treating patients with recurrent AE is critical for lowering morbidity and mortality and improving patient outcomes. In order to successfully manage patients with AE, doctors must have a thorough understanding of the pathophysiology that underlies the condition in order to tailor their treatment to the patient's specific disease. This article contrasts the pathophysiology, clinical manifestation, and treatment of bradykinin-mediated versus histamine-mediated AE in the ED.

## Review

Bradykinin-induced angioedema

General Pathophysiology

The pathophysiology of AE includes an abrupt increase in the permeability of the vessel walls, resulting in local extravasation of plasma, ultimately leading to tissue edema. In bradykinin-induced AE or non-histamine-mediated AE, bradykinin production is enhanced because of improper regulation of the contact pathway, which results in edema. There are three types of bradykinin-mediated AE: histamine-induced AE (HAE), DAE, and acquired AE (AAE) [9].

Kinins are active peptides that are released into the body fluids and tissues through a proteolytic cascade called the kallikrein-kinin cascade, which is also known as the “contact activation pathway.” Factor XII, also called the Hageman factor, is the initiator of the kallikrein-kinin pathway. Though it plays a major role in the contact activation pathway, it is not considered a component of the contact system. Normally, factor XII is autoactivated to factor XIIa in small quantities by contacting the negatively charged tissues and endothelial surfaces or by binding to damaged tissues. During its metabolism, factor XIIa is cleaved to form an active molecule named factor XIIf. Without any opposition, the activation of factor XII to factors XIIa and XIIf results in an increased positive feedback loop. The major inhibitor of factor XIIa is the C1-esterase inhibitor (C1-INH) [10].

**Figure 1:**
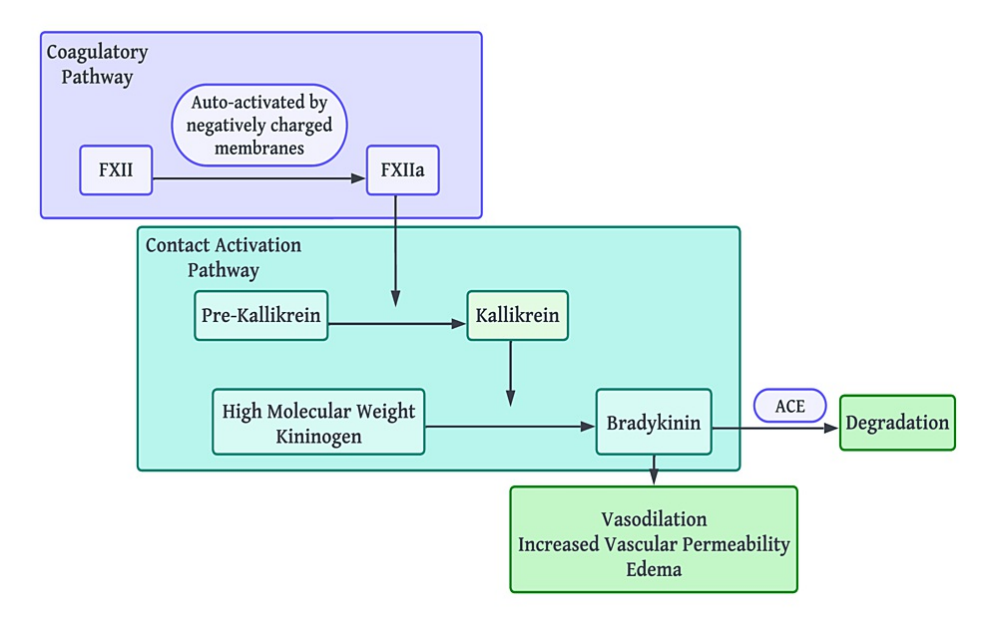
The pathophysiology of bradykinin-mediated angioedema ACE: Angiotensin-converting enzyme. Image credit: Dr. Dilushini Caldera, one of the authors of the current study.

Pre-kallikrein is an inactive precursor of the contact activation pathway. Pre-kallikrein is cleaved by factor XIIa in order to produce an active enzyme called kallikrein. In turn, high molecular weight kininogens are proteolyzed by kallikrein, producing bradykinin. Bradykinin causes vasodilation and an increase in vascular permeability, leading to the development of edema. Bradykinin is rapidly inactivated by angiotensin-converting enzyme (ACE) in endothelial cells [11]. The general pathophysiology of bradykinin-induced angioedema is illustrated in Figure 1.

Bradykinin Receptors

There are two subtypes of bradykinin receptors identified on the cell surfaces, named bradykinin receptor 1 (BKR-1) and bradykinin receptor 2 (BKR-2). BKR-1 is produced in various organs due to tissue injury, and BKR-2 is relatively expressed constantly in healthy tissues. BKR-1 is responsible for immediate acute reactions such as smooth muscle contraction and relaxation as well as more gradual reactions such as collagen synthesis. BKR-1 neuroinflammatory-mediated functions are modulated by interleukins, whereas BKR-2 functions include arterial vasodilation, vasoconstriction, plasma extravasation, and activation of sensory fibers. Both BKR-1 and BKR-2 indirectly use various mechanisms to release prostaglandins, platelet-derived factor, arachidonic acid metabolites, tumor necrosis factor (TNF), interleukin-1 (IL-1), acetylcholine, noradrenaline, and neuropeptides, etc. Furthermore, bradykinin can release three key endothelium-derived vasodilatory mediators such as nitric oxide (NO), prostacyclin (PGI2), and endothelium-derived hyperpolarizing factor (EDHF) [12-14].

Signal Transduction Pathway of Bradykinin

The signaling pathway of BKR-2 on endothelial cells involves phospholipase C (PLC) activation, which leads to hydrolysis of inositol-containing membrane lipids and an increase in inositol 1,4,5-trisphosphate (IP3) and diacylglycerol. PLC activation involves two different isoform families of PLC: PLCβ (there are four isoforms, namely PLCβ1, PLCβ2, PLCβ3, and PLCβ4) and PLCγ (there are two isoforms, namely PLCγ1 and PLCγ2). PLCβ isoforms are activated by G protein, and PLCγ is activated by tyrosine phosphorylation. Bradykinin-stimulated production of IP3 and increased intracellular free Ca2+ concentration are said to be dependent on tyrosine phosphorylation. Therefore, the stimulation of BKR-2 in endothelial cells is followed by the short-term tyrosine phosphorylation of PLCγ1. PLCγ1 interaction with the c-terminal intracellular domain of BKR-2 and increased synthesis of IP3 are both related to phosphorylation, which shows that BKR-2 can bind to other intracellular proteins other than G protein. Therefore, bradykinin-induced IP3 production in endothelial cells may be predominantly attributed to the activation of PLCγ isoforms rather than PLCβ isoforms [15]. Furthermore, studies have shown that bradykinin activates tyrosine kinase 2, resulting in tyrosine phosphorylation and nuclear translocation of the signal transducers and activators of transcription 3 (STAT3) and that in response to bradykinin stimulation, tyrosine kinase 2 and STAT3 form a complex with BKR-2 [16]. Following the stimulation of bradykinin, increased production of NO is mediated by protein kinase A-dependent phosphorylation of endothelial NO synthase [17].

The Physiological Role of Bradykinin

Increased vascular permeability, inflammation, vasodilation, cell proliferation, apoptosis, and the production of the extracellular matrix are a few of the biological outcomes of B2R signaling. Evidence shows that, according to the pathophysiology of some diseases, alteration of BKR-2 activation is either demonstrated as deficient or excessive. High concentrations of bradykinin transiently lower the blood pressure, dilate coronary and peripheral vessels, increase coronary blood flow, improve myocardial metabolism, and have cardioprotective effects due to the activation of BKR-2 on the endothelial cells, which results in increased release of NO, PGI2, EDHF, and a transient rise in tissue plasminogen activators [18,19]. Furthermore, a study showed that the cardioprotective effects of bradykinin can extend toward rendering the myocardium relatively resistant to ischemia [20].

Bronchoconstriction due to the release of inflammatory mediators by bradykinin is considered to be the main reason for dry cough associated with ACE inhibitor use [21]. Studies have shown that bradykinin is linked with the stimulation of bone resorption, and it works as a neuromediator in several cerebral processes, along with the regulation of nociceptive information. Production of pro-inflammatory mediators due to the activation of BKR-2 contributes to inflammation, pain, and hyperalgesia [22-24].

The Pathophysiology of the Three Different Types of Bradykinin-Induced Angioedema

Histamine-induced angioedema (HAE) is an autosomal dominant disorder defined by a deficiency of C1 esterase inhibitor (C1-INH) due to genetic defects, specifically mutations of the Serpin Family G Member 1 (SERP-ING1). HAE is classified into type I, type II, and type III, in which both type I and type II have low C4 levels. HAE type I has low levels of C1-INH associated with loss of function of total plasma C1-INH, whereas type II has normal C1-INH levels but with dysfunctional C1-INH protein [25]. Type III HAE is shown to have a normal level of C1-INH. Studies have shown that there can be an associated mutation of the FXII gene that plays a major role in the activation of the initial kallikrein-kinin pathway, but further studies are needed to know more about the mutations in the FXII gene [26,27]. 

The main roles of C1-INH, which is a serine protease inhibitor, are regulation of the classical complement pathway, contact system, and intrinsic coagulation pathway. At normal physiological concentrations, C1-INH suppresses the activation of the alternative pathway. When C1-INH is not functioning properly, there is an improper activation of the contact pathway as C1-INH is unable to inactivate kallikrein and/or factor XIIa, leading to uncontrolled production of kallikrein and a subsequent increase in the production of bradykinin, which is responsible for acute AE. During the acute attacks of AE, a decrease in the C1-INH levels is linked with spontaneous activation of complement and contact pathways. Therefore, low levels of C2 and C4 show complement system activation, and the presence of high molecular weight kininogen cleavage shows activation of the contact pathway. Low C4 levels are very useful in detecting HAE in laboratory studies, even in asymptomatic cases [28,29].

Acquired AE (AAE) results from nongenetic C1-INH deficiency, typically affecting adults, which is mainly associated with lymphoproliferative disorders, autoimmune, neoplastic, or infectious diseases [30]. Generally, AAE occurs due to increased catabolism of C1-INH that exceeds the host’s capacity to synthesize C1-INH. One molecule of C1-INH inactivates one molecule of its substrate by creating a nonreversible complex with the protease, after which it is removed and destroyed. This proposes a stoichiometric process by which C1INH is depleted when its synthesis is unable to keep up with the activation of its target proteases. People who are affected by an underlying disease that is consistent with the above mechanism lead to continuous activation of the classical pathway, which subsequently depletes C1-INH. AAE is further divided into two subtypes: type I is due to increased consumption of C1-INH and type II is due to inactivation of C1-INH [31]. Type I AAE is frequently seen in patients with rheumatologic disorders and B-cell lymphoproliferative diseases, which clinically manifest after the fourth decade of life. These patients have anti-idiotypic antibodies against immunoglobulins on B cells, which combine and form immune complexes that activate the classical complement pathway. C1-INH is utilized while attempting to neutralize the large amounts of C1 proteases, eventually leading to a deficiency of C1-INH simply because the synthesis cannot keep up with the demand, which ultimately results in AE. Type II AAE results from autoantibodies, typically immunoglobulin G, being produced against C1-INH to inactivate C1-INH [32].

Drug-induced angioedema (DAE) mainly focuses on ACEI-associated AE. ACE is an essential component of the renin-angiotensin-aldosterone system (RAAS). The production of angiotensin II and the degradation of bradykinin are the two main proteolytic functions of ACE. ACE also degrades substance P. Therefore, bradykinin and substance P are linked as mediators in ACEI-induced AE. ACE inhibition decreases the degradation of bradykinin, leading to increased bradykinin concentration, causing AE [33,34]. African American race, female sex, chronic heart failure, coronary heart disease, and smoking are all potential risk factors for developing ACEI-induced AE [35].

Clinical features of bradykinin-induced angioedema

HAE

Patients are usually asymptomatic up to puberty. Although swellings may develop without preceding trauma, more than half of the patients with HAE report some, typically minor, localizing injury, such as dental maneuvers. Other precipitating factors include vigorous exercise, alcohol consumption, emotional stress, and hormonal factors. Coadministration of angiotensin convertase enzyme inhibitors and estrogens is contraindicated in HAE [1]. There is also a transitory prodromal non-pruritic urticarial eruption in some patients. The main sites of cutaneous involvement are the face, hands, arms, legs, genitalia, and buttocks, and the swellings may slowly spread and persist for three to four days. Mucosal involvement is especially feared, and glossal, pharyngeal, or laryngeal involvement can result in respiratory obstruction, asphyxia, and death [1,4,5]. Presentation with abdominal pain and symptoms of intestinal obstruction is also common. Any patient arriving at the ED with these symptoms and many abdominal surgical scars should be evaluated for HAE [1,9]. Pulmonary involvement is infrequent because of high tissue levels of kininases that rapidly inactivate kinin-like peptides. HAE is associated with an increased incidence of many autoimmune disorders and coagulopathies; these include glomerulonephritis, Sjögren syndrome, thyroiditis, and systemic lupus erythematosus [1].

AAE

AAE clinically presents as identical to HAE and is caused by an acquired C1-INH deficiency. These tend to be seen in older age groups without a family history and are commonly associated with lymphoproliferative diseases, such as patients with B-cell lymphoma, the most common associated malignancy, and autoimmune and connective tissue disorders, such as systemic lupus erythematosus [1,4].

DAE

Of all hospital admissions, the most common cause of AE is drugs, especially angiotensin-converting enzyme inhibitors (ACEI), nonsteroidal anti-inflammatory drugs (NSAIDs), and beta-lactam antibiotics [6]. Approximately half of the patients admitted to the hospital for acute AE are patients receiving ACEI therapy [4]. AE caused by ACE inhibitors occurs in approximately 50% of patients within the first week of treatment. The remaining patients may develop symptoms after weeks, months, or even years. It favors the head, neck, lips, mouth, tongue, larynx, pharynx, and subglottal regions without urticaria [1]. Mouth involvement may compromise the airway and cause dysarthria, thus preventing a good case history. Intestinal edema may also occur, causing abdominal pain that may not be accompanied by visible mucocutaneous AE. Therefore, sudden abdominal pain, diarrhea, and vomiting in an adult should promptly be questioned about any recent intake of ACEIs [1].

Histamine-induced angioedema

**Figure 2:**
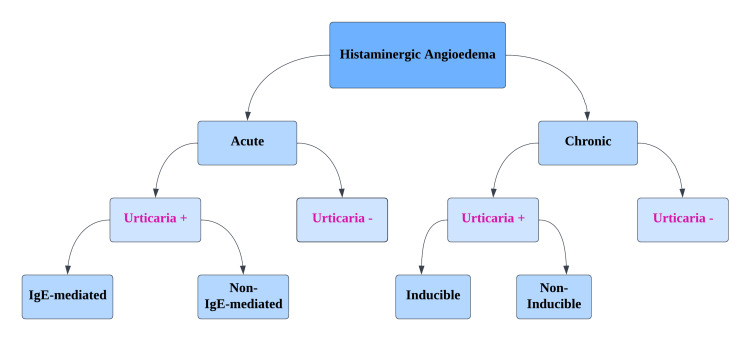
Classification of histamine-mediated angioedema Image credit: Dr. Rahul Jena, one of the authors of the current study.

One of the more frequently encountered conditions in the ED is AE, particularly the histamine-induced variant, accounting for roughly 40%-50% of AE cases in the ER. It is a potentially life-threatening condition, especially without timely intervention [36]. Figure 2 further subdivides histamine-induced AE (HIE) on the basis of a number of criteria, including duration and presence of urticaria [37].

General pathophysiology

**Figure 3:**
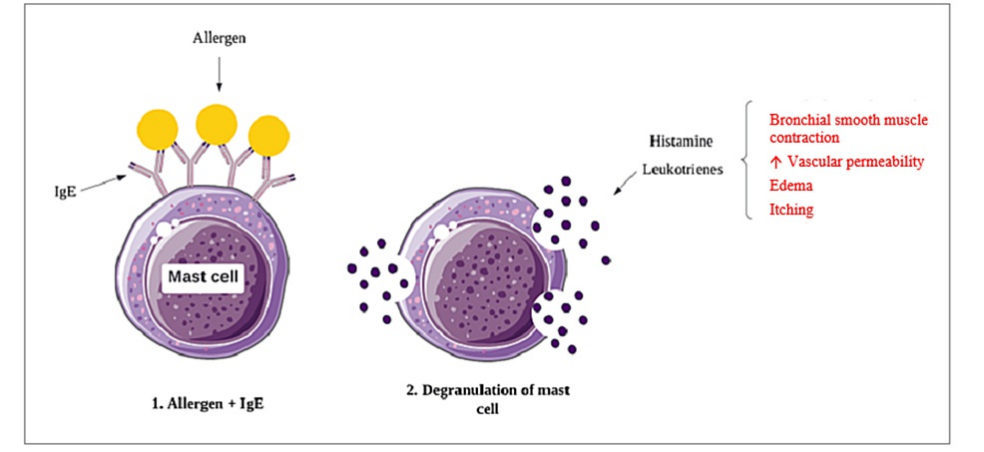
Mechanism of histamine-mediated angioedema Image credit: Dr. Maleesha Jayasinghe, one of the authors of the current study.

HIE usually occurs following allergen exposure and involves a type I hypersensitivity reaction. It usually resolves within 48 hours. Common allergens range from food allergens to insect stings and even exposure to cold (physically induced AE) [3,38]. HIE is potentially life-threatening due to the accompanying laryngeal edema and generalized swelling. This is mediated primarily by the H1 and H2 receptors. On exposure to allergens, there is an increase in the production of immunoglobulin E (IgE) molecules. These are antigen-specific IgE and bind to high-affinity cell receptors on the surface of mast cells and basophils, bringing about ''sensitization.'' When exposed to the same antigen again, bound IgE molecules recognize specific proteins on the Ag, bind to them, and cause "cross-linking." This causes mast cells and basophils to degranulate, resulting in the release of inflammatory mediators such as histamine, tryptase, and chymase. This makes up the "early phase" of the reaction. The late phase of the reaction is brought about by interleukins and growth factors [37,38]. These mediators stimulate the selective histamine receptors, which bring about vasodilation, induce nitric oxide release, and raise blood flow in the submucosal or subcutaneous capillaries, leading to fluid extravasation to the surrounding tissue [37,39]. The same mechanism brings about urticaria, often an accompanying feature of AE, except it takes place in the superficial layers of the skin [37]. Figure 3 depicts the mechanism of histamine-induced AE.

Clinical features

The symptoms of HIE occur mainly on account of the extravasation of intravascular fluid into the surrounding tissue spaces. This leads to localized and transient edema of the deeper skin layers, urogenital system, extremities, and gastrointestinal tract mucosa, which happen to be the two most common sites of involvement [38]. The presentation is often acute in onset. Non-pitting edema and urticarial lesions tend to be the hallmark of histaminergic AEs. Urticarial lesions are blanching, pruritic erythematous papules in the epidermis and are seen in up to 50% of cases of HIE [40]. Other commonly encountered symptoms include laryngeal edema, dyspnea, bronchospasm, wheezing, syncope, and hypotension. Out of these, laryngeal edema is the most dreaded since it is associated with a high mortality rate [37,38]. Hoarseness of voice and stridor are concerning symptoms that should be investigated further [36,41]. The presence of these signs also warrants a detailed respiratory exam to detect wheezing. Involvement of the gastrointestinal tract can range from intermittent abdominal pain to a full-blown acute surgical abdomen. It is also not uncommon for HIE to present with isolated abdominal symptoms without any skin manifestations [3,37]. The biggest assets to the treating physician in the ED are a comprehensive personal and family history and a detailed physical examination. Identification of the mechanism of the underlying edema is critical and truly beneficial to the patient in the long term. Table 1 shows a comparison of the clinical presentation and management of histamine and bradykinin-mediated AE.

**Table 1:** A comparison of the clinical presentation and management of histamine and bradykinin-mediated angioedema HAE: Hereditary angioedema; AAE: Acquired angioedema. Credit: Dr. Omesh Prathiraja, one of the authors of the current study.

Types of angioedema	Histamine-induced	Bradykinin-induced		
Causes	Allergic (anaphylaxis)	HAE	AAE	Drug-induced
Family history	Rare	>75%	None	None
Age of onset	Any	3-20 years	>40 years	None
Severity	Less severe	More severe	More severe	More severe
Onset of swelling	Rapid (minutes)	Slow (hours)	Slow (hours)	Slow (hours)
Recovery of swelling	Faster (12-24 hours)	Slowly (48-72 hours)	Slowly (48-72 hours)	Slowly (48-72 hours)
Prodromal symptoms	None	Frequent	May precede	May precede
Associated symptoms or conditions	Urticaria anaphylaxis (bronchospasm, wheezing, and hypotension)	Abdominal symptoms, no urticaria	Lymphoproliferative and autoimmune	Abdominal symptoms (pain, diarrhea, and vomiting)
Response to standard treatment (epinephrine, antihistamines, and corticosteroids)	Effective	No or minimal response	No or minimal response	No or minimal response

Treatment of bradykinin and histamine-induced angioedema

**Figure 4:**
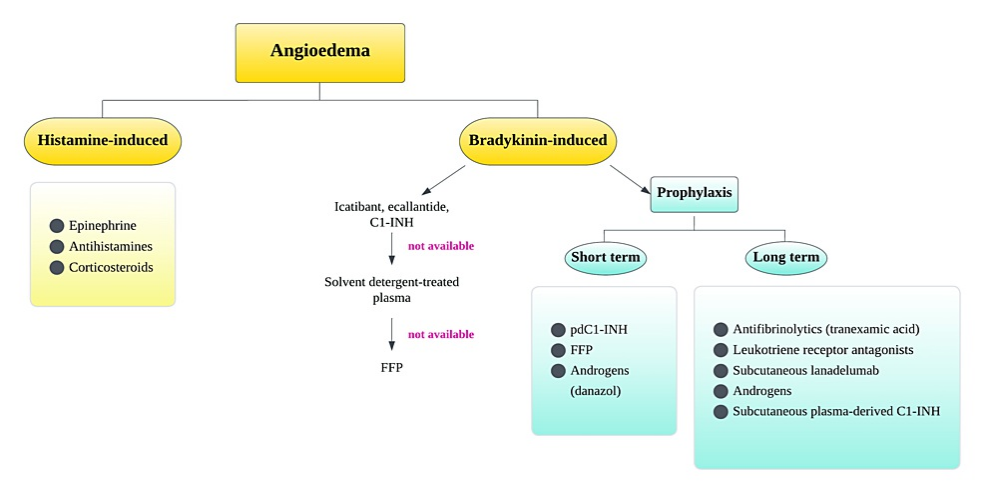
Management of bradykinin and histamine-mediated angioedema C1-INH: C1 esterase inhibitor; pdC1-INH: Plasma-derived C1-inhibitor; FFP: Fresh frozen plasma. Image credit: Dr. Maleesha Jayasinghe, one of the authors of the current study.

Figure 4 shows a summary of the different medications that can be used to treat bradykinin and histamine-induced AE.

Bradykinin-induced angioedema

Hereditary Angioedema

For HAE type I and II, the recommended on-demand treatments are icatibant, ecallantide, and intravenous C1-INH. In the absence of these first-line therapies, the attacks should be treated with solvent-detergent-treated plasma (SDP). In the absence of SDP, the attacks should be treated with fresh frozen plasma (FFP) [42]. HAE attacks are treatable on an "on-demand" basis. Home therapy is recommended because, regardless of the severity of the attack, early treatment reduces the amount of time necessary for the symptoms to resolve. Concentrates of C1INH, such as Ruconest®(Pharming), are indicated for the treatment of all types of HAE attacks in children and adults older than two years. Ruconest is the only available recombinant human C1-INH. It can be self-administered [42,43]. Ecallantide (Kalbitor®; Takeda) is a kallikrein inhibitor that can also be used to treat all types of HAE attacks in HAE type I and type II patients aged 12 years and older. This medication, however, is only approved for use in the United States and a few Latin American countries [42,44]. Because of the serious hypersensitivity reactions, including anaphylaxis, that the medication has been linked to, it should only be administered by a medical professional who has access to the necessary medical care to manage anaphylaxis [42]. Another medication that both adults and children can self-administer for all types of HAE attacks is icatibant (Firazyr®; Takeda). It is a competitive antagonist of the bradykinin B2 receptor that prevents the binding of bradykinin to its receptor. One injection of icatibant resolves the majority of attacks. Despite the possibility of transient local injection site reactions (erythema, wheal, pruritus, and burning sensation), icatibant is generally safe and well tolerated. There were no reports of allergic reactions [42].

Acquired Angioedema

Most AAE patients who receive this treatment report success, but not all do. Some AAE patients require higher doses of plasma-derived C1-INH or become progressively nonresponsive to it. There is no known therapeutic alternative to plasma-derived C1-INH for life-threatening attacks because no other therapy for AE attacks has been widely used in patients with AAE. During an emergency, invasive techniques have been administered to nonresponsive patients to maintain the patency of their upper airways. Additionally, ecallantide and icatibant are used in the management of AAE [45]. In the preventative care of AAE, long-term therapy with antifibrinolytics has demonstrated superior efficacy to attenuated androgens [32]. According to a recent study by Kesh et al., tranexamic acid (TXA) treatment for AAE can be used to prevent AE attacks. However, potentially harmful consequences remain a concern, emphasizing the need for new alternatives. Eleven out of the 13 patients in this study were receiving TXA as prophylaxis. About 97%, 86%, and 99% reduction in AE episodes was noted at 1, 12, and 24 months following TXA treatment [46]. In addition, C1-INH can also be given for long-term prevention in selective patients with two or more attacks per week [32].

Drug-Induced Angioedema

Although ACEIs are the most prevalent class of medications involved with the development of DAE, only 0.5% of individuals who are taking ACEIs will suffer this side effect compared to 5.5% of the black population. Aspirin and other NSAIDs can also potentially trigger AE. During the initial week of therapy, attacks are common. When obtaining a patient's medical history, it is essential to search for information implicating these causative agents. Attacks may subside without treatment beyond the removal of the offending substance. However, in severe cases, intramuscular adrenaline and endotracheal intubation may be required [47].

Histamine-Induced Angioedema

Elimination of the causative agent is the first step in treating acute histamine-induced AE. This form of AE may occur during anaphylaxis. Consequently, it is crucial to prioritize airway stabilization and prevent circulatory collapse [48]. Flexible laryngoscopy may be utilized to identify the extent of airway involvement by AE and, consequently, the need for intubation [49]. In contrast to non-histamine-related types of AE, which are resistant to standard treatments and frequently necessitate airway support owing to respiratory distress, histamine-induced AE responds effectively to medications, reducing the risk of intubation [48].

Patients must receive epinephrine, steroids, antihistamines, and IV fluids [9,48]. Histamine-mediated AE should be assumed if there is any possibility of urticaria-associated AE or anaphylaxis or if the precise underlying cause of the AE is unknown [38].

Epinephrine needs to be given intramuscularly (IM) right away if systemic anaphylaxis is suspected. Adults should receive 0.3-0.5 mg of intramuscularly administered epinephrine (1 mg/mL or 1:1000 dilution) in the thigh, while children should receive 0.01 mg/kg (up to 0.03 mg) [9]. If necessary, epinephrine may be administered every 20 minutes to once every four hours until symptoms are under control [50]. Patients who require multiple intramuscular epinephrine injections should be considered for IV epinephrine administration, with doses ranging from 1 to 4 micrograms (mcg) per minute [38].

To treat acute histamine-mediated AE, diphenhydramine that has a rapid onset of action can be administered orally, intramuscularly, or intravenously. If anaphylaxis or respiratory distress is a major concern, epinephrine should always be administered instead. The addition of H2 antagonists to H1 antagonists can also be considered as this combination may aid in the prevention of pruritus, urticaria, and hypotension. However, there is not enough evidence to support the use of this latter intervention in an acute allergic emergency [51].

To reduce inflammatory mediators in HAE, steroids such as IV methylprednisolone (125 mg) can be used. Generally, this drug takes four to six hours to take effect after administration [38].

Limitations

This study relies on a survey of open-access research journals published over the past decade; as a result, we may have omitted important material from paid full-text journals and research articles published prior to 2015. In addition, as our analysis is limited to English-language studies, we may have overlooked studies that are published in other languages.

## Conclusions

Due to its ability to cause airway obstruction, AE poses a challenge for ED physicians. In clinical practice, it is difficult to differentiate histamine-induced AE from bradykinin-mediated AE despite its relatively rapid onset and association with urticaria. AE brought on by histamine can be treated with epinephrine, antihistamines, and steroids. If the patient does not respond to these treatments, bradykinin-induced AE must be suspected and appropriately treated. C1-INH, icatibant, and ecallantide have a place in the treatment of bradykinin-mediated AE. More research is required to distinguish between these two types of AE as they are treated differently in emergency settings.

## References

[1] Kaplan AP (2008). Angioedema. World Allergy Organ J.

[2] Saini SS (2014). Urticaria and angioedema. Middleton's Allergy (Eighth Edition).

[3] Frigas E, Nzeako UC (2002). Angioedema. Pathogenesis, differential diagnosis, and treatment. Clin Rev Allergy Immunol.

[4] Gülbahar O (2021). Angioedema without wheals: a clinical update. Balkan Med J.

[5] Hébert J, Boursiquot JN, Chapdelaine H (2022). Bradykinin-induced angioedema in the emergency department. Int J Emerg Med.

[6] Zingale LC, Beltrami L, Zanichelli A, Maggioni L, Pappalardo E, Cicardi B, Cicardi M (2006). Angioedema without urticaria: a large clinical survey. CMAJ.

[7] Tai S, Mascaro M, Goldstein NA (2010). Angioedema: a review of 367 episodes presenting to three tertiary care hospitals. Ann Otol Rhinol Laryngol.

[8] Rye Rasmussen EH, Bindslev-Jensen C, Bygum A (2012). Angioedema--assessment and treatment. Tidsskr Nor Laegeforen.

[9] Bernstein JA, Moellman J (2012). Emerging concepts in the diagnosis and treatment of patients with undifferentiated angioedema. Int J Emerg Med.

[10] Nzeako UC, Frigas E, Tremaine WJ (2001). Hereditary angioedema: a broad review for clinicians. Arch Intern Med.

[11] Depetri F, Tedeschi A, Cugno M (2019). Angioedema and emergency medicine: from pathophysiology to diagnosis and treatment. Eur J Intern Med.

[12] Sriramula S (2020). Kinin B1 receptor: a target for neuroinflammation in hypertension. Pharmacol Res.

[13] Regoli D, Jukic D, Gobeil F, Rhaleb NE (1993). Receptors for bradykinin and related kinins: a critical analysis. Can J Physiol Pharmacol.

[14] Busse R, Fleming I (2003). Regulation of endothelium-derived vasoactive autacoid production by hemodynamic forces. Trends Pharmacol Sci.

[15] Venema VJ, Ju H, Sun J, Eaton DC, Marrero MB, Venema RC (1998). Bradykinin stimulates the tyrosine phosphorylation and bradykinin B2 receptor association of phospholipase C gamma 1 in vascular endothelial cells. Biochem Biophys Res Commun.

[16] Ju H, Venema VJ, Liang H, Harris MB, Zou R, Venema RC (2000). Bradykinin activates the Janus-activated kinase/signal transducers and activators of transcription (JAK/STAT) pathway in vascular endothelial cells: localization of JAK/STAT signalling proteins in plasmalemmal caveolae. Biochem J.

[17] Bae SW, Kim HS, Cha YN, Park YS, Jo SA, Jo I (2003). Rapid increase in endothelial nitric oxide production by bradykinin is mediated by protein kinase A signaling pathway. Biochem Biophys Res Commun.

[18] Blaes N, Girolami JP (2013). Targeting the 'Janus face' of the B2-bradykinin receptor. Expert Opin Ther Targets.

[19] Smith D, Gilbert M, Owen WG (1985). Tissue plasminogen activator release in vivo in response to vasoactive agents. Blood.

[20] Leesar MA, Stoddard MF, Manchikalapudi S, Bolli R (1999). Bradykinin-induced preconditioning in patients undergoing coronary angioplasty. J Am Coll Cardiol.

[21] Fuller RW, Dixon CM, Cuss FM, Barnes PJ (1987). Bradykinin-induced bronchoconstriction in humans. Mode of action. Am Rev Respir Dis.

[22] Lerner UH (1994). Regulation of bone metabolism by the kallikrein-kinin system, the coagulation cascade, and the acute-phase reactants. Oral Surg Oral Med Oral Pathol.

[23] Couture R, Harrisson M, Vianna RM, Cloutier F (2001). Kinin receptors in pain and inflammation. Eur J Pharmacol.

[24] Dray A (1997). Kinins and their receptors in hyperalgesia. Can J Physiol Pharmacol.

[25] Reshef A, Leibovich I, Goren A (2008). Hereditary angioedema: new hopes for an orphan disease. Isr Med Assoc J.

[26] Bork K, Wulff K, Meinke P, Wagner N, Hardt J, Witzke G (2011). A novel mutation in the coagulation factor 12 gene in subjects with hereditary angioedema and normal C1-inhibitor. Clin Immunol.

[27] Bork K, Gül D, Dewald G (2006). Hereditary angio-oedema with normal C1 inhibitor in a family with affected women and men. Br J Dermatol.

[28] Davis AE 3rd (2005). The pathophysiology of hereditary angioedema. Clin Immunol.

[29] Banerji A (2011). Hereditary angioedema: classification, pathogenesis, and diagnosis. Allergy Asthma Proc.

[30] Agostoni A, Aygören-Pürsün E, Binkley KE (2004). Hereditary and acquired angioedema: problems and progress: proceedings of the third C1 esterase inhibitor deficiency workshop and beyond. J Allergy Clin Immunol.

[31] Zuraw BL, Bernstein JA, Lang DM (2013). A focused parameter update: hereditary angioedema, acquired C1 inhibitor deficiency, and angiotensin-converting enzyme inhibitor-associated angioedema. J Allergy Clin Immunol.

[32] Patel G, Pongracic JA (2019). Hereditary and acquired angioedema. Allergy Asthma Proc.

[33] Lefebvre J, Murphey LJ, Hartert TV, Jiao Shan R, Simmons WH, Brown NJ (2002). Dipeptidyl peptidase IV activity in patients with ACE-inhibitor-associated angioedema. Hypertension.

[34] Dicpinigaitis PV (2006). Angiotensin-converting enzyme inhibitor-induced cough: ACCP evidence-based clinical practice guidelines. Chest.

[35] Morimoto T, Gandhi TK, Fiskio JM (2004). An evaluation of risk factors for adverse drug events associated with angiotensin-converting enzyme inhibitors. J Eval Clin Pract.

[36] Moellman JJ, Bernstein JA, Lindsell C (2014). A consensus parameter for the evaluation and management of angioedema in the emergency department. Acad Emerg Med.

[37] Busse PJ, Smith T (2017). Histaminergic angioedema. Immunol Allergy Clin North Am.

[38] Long BJ, Koyfman A, Gottlieb M (2019). Evaluation and management of angioedema in the emergency department. West J Emerg Med.

[39] Durán WN, Breslin JW, Sánchez FA (2010). The NO cascade, eNOS location, and microvascular permeability. Cardiovasc Res.

[40] Zuraw BL (2008). Clinical practice. Hereditary angioedema. N Engl J Med.

[41] Bork K (2010). Recurrent angioedema and the threat of asphyxiation. Dtsch Arztebl Int.

[42] Maurer M, Magerl M, Betschel S (2022). The international WAO/EAACI guideline for the management of hereditary angioedema-the 2021 revision and update. Allergy.

[43] Longhurst HJ, Gonçalo M, Godse K, Ensina LF (2021). Managing chronic urticaria and recurrent angioedema differently with advancing age. J Allergy Clin Immunol Pract.

[44] (2022). Application to market ecallantide in Europe is withdrawn. https://www.medscape.com/viewarticle/753894.

[45] Cicardi M, Zanichelli A (2010). Acquired angioedema. Allergy Asthma Clin Immunol.

[46] Kesh S, Singh U, Bernstein JA (2022). Longitudinal experience with treatment of acquired angioedema using tranexamic acid. Allergy Asthma Proc.

[47] (2006). Causes and management of drug‐induced angioedema. https://wchh.onlinelibrary.wiley.com/doi/pdf/10.1002/psb.358.

[48] James C, Bernstein JA (2017). Current and future therapies for the treatment of histamine-induced angioedema. Expert Opin Pharmacother.

[49] Byrd JB, Adam A, Brown NJ (2006). Angiotensin-converting enzyme inhibitor-associated angioedema. Immunol Allergy Clin North Am.

[50] Lieberman P, Nicklas RA, Oppenheimer J (2010). The diagnosis and management of anaphylaxis practice parameter: 2010 update. J Allergy Clin Immunol.

[51] Lin RY, Curry A, Pesola GR (2000). Improved outcomes in patients with acute allergic syndromes who are treated with combined H1 and H2 antagonists. Ann Emerg Med.

